# An Analytical Model for the Distributions of Velocity and Discharge in Compound Channels with Submerged Vegetation

**DOI:** 10.1371/journal.pone.0130841

**Published:** 2015-07-10

**Authors:** Beihan Jiang, Kejun Yang, Shuyou Cao

**Affiliations:** 1 College of Civil Engineering, Fuzhou University, Fuzhou, Fujian, China; 2 State Key Laboratory of Hydraulics and Mountain River Engineering, Sichuan University, Chengdu, Sichuan, China; Centro de Investigacion Cientifica y Educacion Superior de Ensenada, MEXICO

## Abstract

Based on the momentum transfer theory, an analytical model is proposed for the velocity and discharge distributions in compound channels with submerged vegetation on the floodplain. The partially vegetated channel was divided into three sub-regions, i.e. the main channel region, the floodplain region with submerged vegetation and the floodplain region without vegetation. For each region, the force balance relationship was established, and the momentum transfer between different regions was presented. Verification by the experimental data and comparison with the traditional method shows that the proposed method is capable of predicting for the velocity and discharge distributions in compound channels with submerged vegetation and is superior to the conventional method. The results also show that when the momentum transfer between different regions is ignored, the computed discharge will be much lager than the measured data, and the error increases with the discharge, especially in the floodplain region.

## Introduction

A compound channel consisting of a main channel and one or two floodplains yields complex flow conditions. The shear layer, which develops at the interaction region between the main channel and the floodplain due to momentum transfer from the flows in the main channel to the floodplain, together with the secondary flow, influences significantly the mean flow and turbulence statistics [1]. In addition, if the floodplain is vegetated, which is under natural conditions, flow behavior becomes more complex. The vegetation considerably improves river environment and geomorphic stability but it influences the channel resistance and reduces the flood carrying capacity extremely by generating additional drag [2, 3, 4]. This increases bed roughness, reduces mean velocity in the floodplain and enhances the velocities difference between the main channel and the floodplain. As a result, strong eddies and violent transverse mixings are produced at the interface. Consequently, conveyance estimation methods appropriate to single channels are not accurate when applied to overbank flows; they may lead to either over-estimation of discharge capacity, which is dangerous for the people and properties, or to under-estimation, which may cause overdesign and waste of project cost [5]. By this, knowledge of the velocity distribution of the main channels and floodplains may be very helpful in the prediction of flow capacity and, consequently, stage-discharge relationships. These parameters are key factors in controlling floods and in designing artificial waterways.

Many researchers have estimated the discharge capacity of compound channels. The main methods of conveyance estimation are as follows, (1) the single-channel method (SCM), (2) the divided channel method (DCM) [6, 7, 8], (3) the momentum transfer and the apparent shear stress method (ASSM), where the momentum transfer at the main channel-floodplain interface can be described as an apparent shear stress [9, 10], and shear stress at the interface of the main channel and the floodplain is assumed as the dominating stress [11, 12], (4) the coherence method (COHM), which is one-dimensional approach based on the 'coherence' concept proposed by Ackers [13–15], (5) comprehensive roughness method (CRM), where the roughness is integrated taking the variation of wetted perimeter into account. Prinos and Townsend [16] pointed out the single-channel method would lead to underestimation of discharge, and divided channel method would lead to overestimation, especially at low relative depth.

As for the vegetated single and compound channels, some investigations into flow characteristics and its modeling method have been undertaken. Huai et al. [17] investigated the mechanism of energy balance in an open-channel flow with submerged vegetation. Huang et al. [18] had investigated the velocity profile distribution and the significant contribution of roughness coefficient on the discharge distribution. Shiono and Knight obtained an analytical solution to predict the depth-averaged velocity distributions and then gives prediction for the discharge capacity by laterally integrating depth-averaged velocity [1,19, 20]. Sun and Shiono [21] investigated the flow and turbulence structures in compound channels with one-line emergent vegetation along the floodplain edge and used friction factor obtained by regression analyses to predict the stage-discharge curve. Huai et al. [22–26] proposed analytical models for predicting the lateral and vertical distributions of mean streamwise velocity in single channels, by considering the effects of double-layered rigid vegetation, submerged vegetation, and suspended vegetation, respectively. Some scholars (e.g. Choi and Kang [27], Fisher-Antze et al. [28]) considered the drag force as an additional force sink term, which is implemented into the momentum equations to numerically simulate flow characteristics in vegetated channels. Huai et al. [29,30], Tang and Knight [31, 32], Liu et al. [33] established the governing equations of flow in compound channels with vegetated floodplains and gave good predictions for the lateral distributions of depth-averaged velocity and boundary shear stress for the type of channels.

As it is seen, the majority of previous efforts to estimate the velocity and discharge in compound channels have focused on the floodplains with emergent vegetation. However, unfortunately, there are barely studies on compound channels flow with partially submerged vegetation. In this study, based on the momentum transfer theory [34], an attempt is made to propose a relatively simple, useful mathematical model for the predictions of average flow velocities and discharges for each channel region. The accuracy of the analytical model is evaluated using data from Yang et al. [35] and Sun [36].

## Mathematical Model

### Vegetation resistance

**Fig 1:**
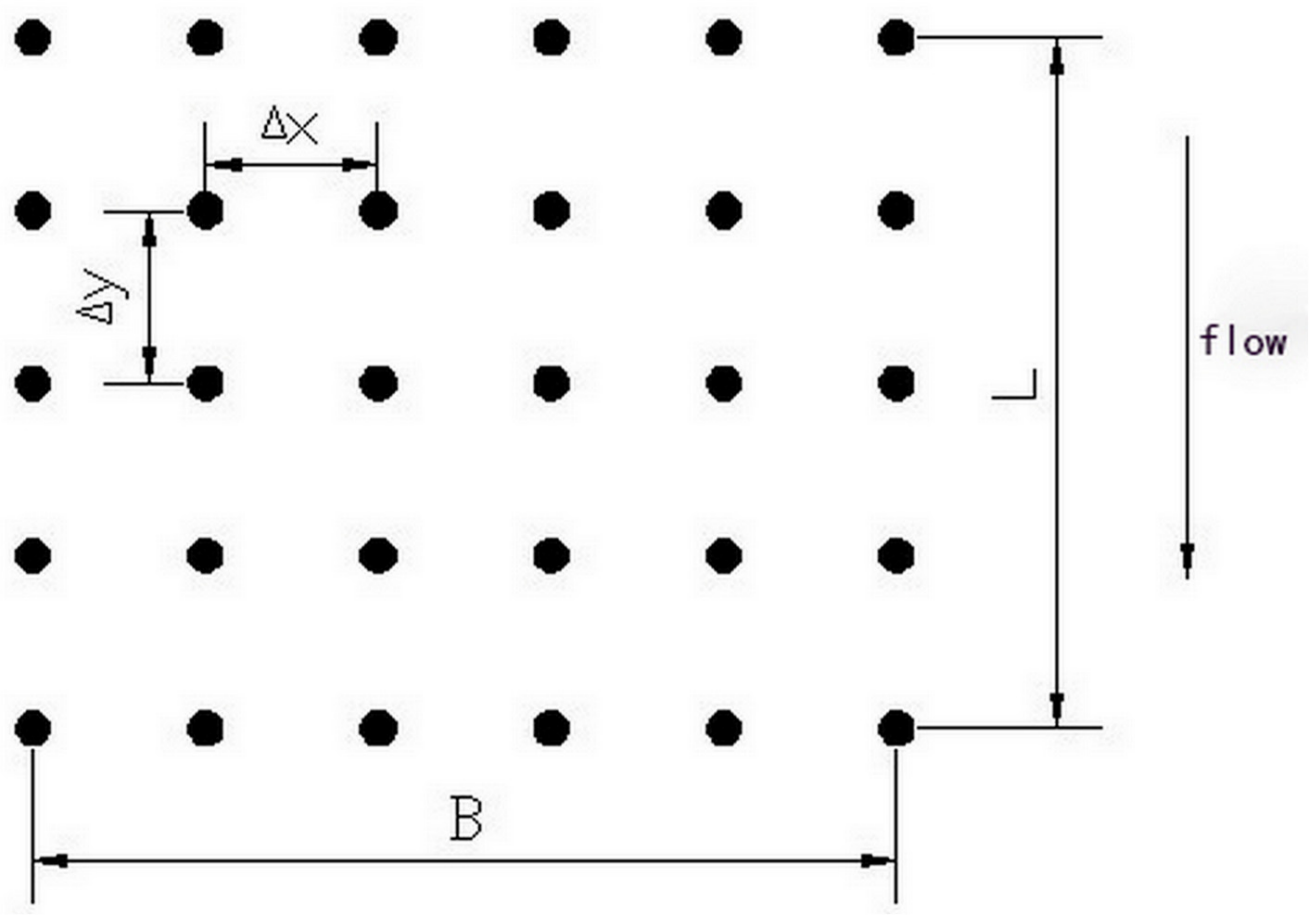
Vegetation distribution of the cross section. The cylinders of simulated vegetation are uniformly distributed in the streamwise (x) and in the transverse (y) directions.

For flow around a cylinder, according to its forming reason, the forces acting on the cylinder can be divided into deformation resistance, frictional resistance, form resistance and induced resistance and so on. With large Reynolds number, the main resistance on the vegetation is form resistance for the breakaway detour flow. The cylinders of simulated vegetation are uniformly distributed in the streamwise (*x*) and in the transverse (*y*) directions as shown in Fig 1, in which L and B is the longitudinal length and transverse width, respectively; △x and △y is the average longitudinal length and average transverse width between the vegetation. The vegetation drag term used the classical formula for flow around a cylinder,

$$F_{v}=\frac{1}{2}N\rho C_{D}A_{v}U^{2}BL$$ (1)

where *N* (=1/Δ*x*Δ*y*) is the density of the vegetation, *C*_*D*_ is drag coefficient, Tanino and Nepf [34] showed that the drag coefficient (*C*_*D*_) is related to rod Reynolds number Re_*D*_ (= *U*_d_ *D*/ν), D is the diameter of the rod. *A*_*v*_ is the projected area of the vegetation in the streamwise direction per unit volume.

### Hydraulic calculation of the compound channels with submerge-vegetation floodplains

The flow in the floodplain will come into the main channel at the beginning of the compound channels with vegetated floodplains, but after a long distance, that is, in the channels after the end of floodplain flow coming into main channels, the flow will exchange between the main channel and the floodplains, which form the relatively low velocity in the floodplains. At this time, the flow can be generalized as the uniform flow on the time-averaged, namely the force reached a balance between the main channels and the floodplains. The following formula derivation is based on the assumption of uniform and steady flow.

In a single channel for steady uniform flow, the lateral velocity gradient for depth-average velocity does not exist basically, except for the channel side slope zone. Because there is no relative movement between neighboring water, especially for the wide-shallow reaches, the lateral momentum exchange can be considered not existing. However for the compound channel, there is lateral velocity gradient for depth-average velocity, which causes the momentum exchange between adjacent regions, especially in the interaction area between main channel and floodplain, leading to lateral apparent shear stress in these areas.

Identically, the submerged vegetation flow can be divided into two layers: the upper one which is characterized by the flow above the top of vegetation and the lower one by the flow through the vegetation [37]. The mean velocities differ greatly between two regions. In the lower vegetated flow region, affected by the vegetation drag, the velocity is much smaller than in the upper region. So there is momentum exchange in the interface between upper and lower regions, and vertical apparent shear stress is generated from that. Wu [38] pointed out the turbulence characteristics was anisotropy for the submerged vegetated flow, and the maximum momentum exchange was around the top of vegetation. The vertical apparent shear stress cannot be neglected in the vegetated region.

**Fig 2:**
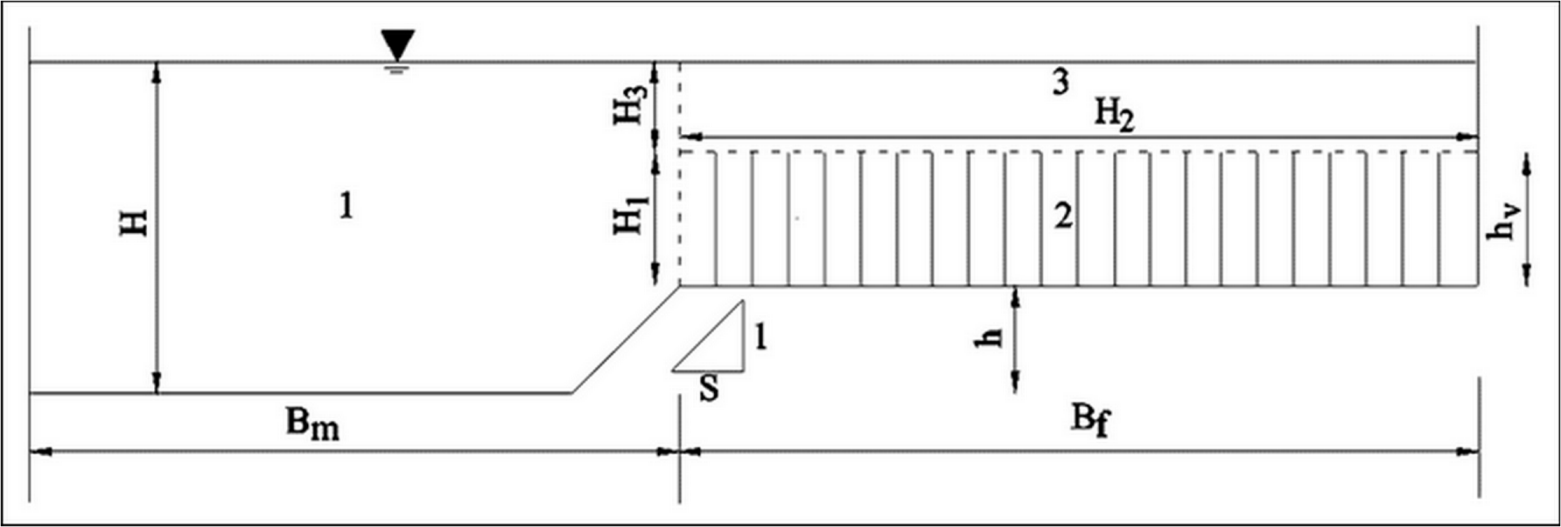
Cross section of submerged vegetated compound channel. The submerged-vegetation compound channel is divided into three sub-regions, namely the main channel region (Region 1), the floodplain region with submerged vegetation (Region 2) and the floodplain region without vegetation (Region 3).

As shown in Fig 2, the submerged-vegetation compound channel is divided into three sub-regions, namely the main channel region (Region 1), the floodplain region with submerged vegetation (Region 2) and the floodplain region without vegetation (Region 3). For the uniform and steady flow, the force balance relationships can be set up for a given water control volume, whose longitudinal length is 1.

At the interface of a main channel and a floodplain, the transfer of momentum takes in the form of an apparent shear stress. Hence, for the control volume in the main channel region, the forces acting on the control volume are the gravity, boundary shear force and the apparent shear force between the main channel and the floodplain. The force balance relationship is,

$$W_{1}\text{sin}\;\theta =P_{1}\tau_{1}+H_{1}\tau_{a1}+H_{2}\tau_{a2}$$ (2)

where W_1_ is the gravity of the water in the control volume of Region 1, P_l_ is the wetted perimeter of the main channel, sin *θ* (= *S*_0_) is the bed slope gradient, *τ*_1_ is the boundary shear stress, *τ*_*a*1_ is the apparent shear stress between Region 1 and Region 2, *τ*_*a*2_ is the apparent shear stress between Region 1 and Region 3, *H*_1_(= *h*_*v*_) is the flow height of the interaction surface between the Region 1 and the Region 2, and *H*_2_(= *H* − *h* − *h*_*v*_) is the height of the interaction surface between Region 1 and Region 3.

As for the control volume of Region 2, besides the gravity, the boundary shear force, vegetation drag force and the apparent shear forces due to the momentum transfer between Region 1and Region 2, and that between Region 2 and Region 3, Vegetation can significantly reduce the mean velocity on the Region 2 by increasing the flow resistance, leading to a similar shear layer between the vegetated floodplain flow and non-vegetated floodplain flow. Hence, the force balance relationship in Region 2, may be presented by the following equation,

$$\delta W_{2}\;\text{sin}\;\theta =P_{2}\tau_{2}-H_{1}\tau_{a1}-H_{3}\tau_{a3}+\frac{1}{2}N\rho C_{D}A_{v}V_{2}^{\;2}H_{3}$$ (3)

where W_2_ is the gravity of the water in the control volume of Region 2, P_2_ is the wetted perimeter of Region 2, *τ*_2_ is the boundary shear stress, *τ*_*a*3_ is the apparent shear stress between Region 2 and Region 3, *δ* (= 1 − *nA*_*v*_) expresses the blockage effect of the vegetation, n is the volumetric vegetation density, *V*_2_ is the average velocity of Region 2, *H*_3_ (= *B*_*f*_) is the width of the interaction surface between Region 2 and Region 3.

As for the control volume of Region 3, the force balance relationship is,

$$W_{3}\;\text{sin}\;\theta =P_{3}\tau_{3}-H_{2}\tau_{a2}+H_{3}\tau_{a3}$$ (4)

where W_3_ is the gravity of the water in the control volume of Region 3, P_3_ is the wetted perimeter of Region 3.

The force balance relationship of the whole cross section can be obtained by adding Eqs (2–4) together,

$$(W_{1}+W_{2}+W_{3})\text{sin}\;\theta =P_{1}\tau_{1}+P_{2}\tau_{2}+P_{3}\tau_{3}+\frac{1}{2}N\rho C_{D}A_{v}U^{2}H_{3}$$ (5)

Then substituting *W*_1_ = *γA*_1_ *L*, *W*_2_ = *γA*_2_ *L* and *W*_3_ = *γA*_3_ *L* into Eqs (2–4),

$$\tau_{1}=\gamma R_{1}S_{0}-\frac{H_{1}}{P_{1}}\tau_{a1}-\frac{H_{2}}{P_{1}}\tau_{a2}$$ (6)

$$\tau_{2}=\delta \gamma R_{2}S_{0}+\frac{H_{1}}{P_{2}}\tau_{a1}+\frac{H_{3}}{P_{2}}\tau_{a3}-\frac{N\rho C_{D}A_{v}V_{2}^{\;2}H_{3}}{2P_{2}}$$ (7)

$$\tau_{3}=\gamma R_{3}S_{0}+\frac{H_{2}}{P_{3}}\tau_{a2}-\frac{H_{3}}{P_{3}}\tau_{a3}$$ (8)

To solve Eqs (6–8), a complementary relational expression is required. A mixing zone exists between the main channel and the floodplain because of the momentum transfer. The flow in this region can be taken as free shear turbulence [15], for which there is a self-preserving hypothesis, namely in the development process of turbulence, the distributions of each physical quantities can maintain some properties, and only the length scale and the time scale change along. The experimental data of SERC-FCF showed that the lateral distributions of streamwise velocity in the interaction region between the main channel and floodplain are similar. According to Prandtl momentum transfer theory [34], the apparent shear stress may be expressed as,

$$\tau_{a}=\rho \varepsilon \frac{\partial u}{\partial y}$$ (9)

where *ε* is the eddy viscosity, and y is the lateral direction. The similarity of velocity distributions gives the expression,

$$\varepsilon =a_{0}uL$$ (10)

where *a*_0_ is a proportionality coefficient, *u* and *L* are the characteristic velocity and length in the mixing zone, Liu and Dong [39] assumed that,

$$L\propto b_{z}$$ (11)

$$\frac{\partial u}{\partial y}\propto \frac{V_{1}-V_{2}}{b_{z}}$$ (12)

$$u\propto \frac{V_{1}+V_{2}}{2}$$ (13)

where *b*_*z*_ is the characteristic width. Substituting Eqs (11–13) into Eqs (9) and (10) gives,

$$\tau_{a}=\alpha \rho \frac{V_{1}^{\;2}-V_{2}^{\;2}}{2}$$ (14)

where *α* is defined as the momentum transfer coefficient, and *V*_1_ and *V*_2_ are the mean velocities in the main channel and the floodplain region with vegetation. The uniform flow gives $\tau =f\frac{\rho }{8}V^{2}$ and $f=\frac{8g}{C^{2}}$, so

$$\tau_{1}=\gamma \frac{V_{1}^{\;2}}{C_{1}^{\;2}}$$ (15)

$$\tau_{2}=\gamma \frac{V_{2}^{\;2}}{C_{2}^{\;2}}$$ (16)

$$\tau_{3}=\gamma \frac{V_{3}^{\;2}}{C_{3}^{\;2}}$$ (17)

where *C* is the Chezy coefficient, which can be obtained by Manning equation. Substituting Eqs (14–16) into Eqs (7–9) gives,

$$\frac{V_{1}^{\;2}}{C_{1}^{\;2}}=\frac{A_{1}}{P_{1}}S_{0}-\alpha_{1}\frac{H_{1}}{2P_{1}g}(V_{1}^{\;2}-V_{2}^{\;2})-\alpha_{2}\frac{H_{2}}{2P_{1}g}(V_{1}^{\;2}-V_{3}^{\;2})$$ (18)

$$\frac{V_{2}^{\;2}}{C_{2}^{\;2}}=\delta \frac{A_{2}}{P_{2}}S_{0}+\alpha_{1}\frac{H_{1}}{2P_{2}g}(V_{1}^{\;2}-V_{2}^{\;2})+\alpha_{3}\frac{H_{3}}{2P_{2}g}(V_{3}^{\;2}-V_{2}^{\;2})-\frac{N\rho C_{D}A_{v}V_{2}^{\;2}H_{3}}{2P_{2}g}$$ (19)

$$\frac{V_{3}^{\;2}}{C_{3}^{\;2}}=\frac{A_{3}}{P_{3}}S_{0}+\alpha_{2}\frac{H_{2}}{2P_{3}g}(V_{1}^{\;2}-V_{3}^{\;2})-\alpha_{3}\frac{H_{3}}{2P_{3}g}(V_{3}^{\;2}-V_{2}^{\;2})$$ (20)

Eqs (18–20) is a system of linear equations with respect to the square of velocity in each sub-region, $V_{1}^{2}$, $V_{2}^{2}$ and $V_{3}^{2}$. Setting,

$$\frac{1}{C_{1}^{\;2}}=I_{1},\frac{A_{1}}{P_{1}}S_{0}=J_{1},\alpha_{1}\frac{H_{1}}{2P_{1}g}=K_{1},\alpha_{2}\frac{H_{2}}{2P_{1}g}=M_{1}$$ (21)

$$\frac{1}{C_{2}^{\;2}}=I_{2},\delta \frac{A_{2}}{P_{2}}S_{0}=J_{2},\alpha_{1}\frac{H_{1}}{2P_{2}g}=K_{2},\alpha_{3}\frac{H_{3}}{2P_{2}g}=M_{2},\frac{N\rho C_{D}A_{v}H_{3}}{2P_{2}g}=N_{2}$$ (22)

$$\frac{1}{C_{3}^{\;2}}=I_{3},\frac{A_{3}}{P_{3}}S_{0}=J_{3},\alpha_{2}\frac{H_{2}}{2P_{3}g}=K_{3},\alpha_{3}\frac{H_{3}}{2P_{3}g}=M_{3}$$ (23)

Eqs (18–20) are simplified into,

$$I_{1}V_{1}^{\;2}=J_{1}-K_{1}(V_{1}^{\;2}-V_{2}^{\;2})-M_{1}(V_{1}^{\;2}-V_{3}^{\;2})$$ (24)

$$I_{2}V_{2}^{\;2}=J_{2}+K_{2}(V_{1}^{\;2}-V_{2}^{\;2})+M_{2}(V_{3}^{\;2}-V_{2}^{\;2})-N_{2}V_{2}^{\;2}$$ (25)

$$I_{3}V_{3}^{\;2}=J_{3}+K_{3}(V_{1}^{\;2}-V_{3}^{\;2})-M_{3}(V_{3}^{\;2}-V_{2}^{\;2})$$ (26)

Setting,

$$\frac{1}{I_{1}+K_{1}+M_{1}}=Q$$ (27)

$$\frac{1}{I_{2}+K_{2}+M_{2}+N_{2}}=R$$ (28)

$$\frac{1}{I_{3}+K_{3}+M_{3}}=\text{T}$$ (29)

Eqs (24–26) are formulized as,

$$V_{1}^{\;2}=J_{1}Q-K_{1}QV_{2}^{\;2}+M_{1}QV_{3}^{\;2}$$ (30)

$$V_{2}^{\;2}=J_{2}R+K_{2}RV_{1}^{\;2}+M_{2}RV_{3}^{\;2}$$ (31)

$$V_{3}^{\;2}=J_{3}T+K_{3}TV_{1}^{\;2}+M_{3}TV_{2}^{\;2}$$ (32)

The analytical expression of the velocity of each sub-region can be obtained by solving simultaneous Eqs (30–32),

$$V_{1}=\sqrt{\frac{\left(J_{1}Q+M_{1}QJ_{3}T\right)\left(1-M_{2}RM_{3}T\right)+\left(K_{1}Q+M_{1}QM_{3}T\right)\left(J_{2}R+M_{2}RJ_{3}T\right)}{\left(1-M_{1}QK_{3}T\right)\left(1-M_{2}RM_{3}T\right)-\left(K_{1}Q+M_{1}QM_{3}T\right)\left(K_{2}R+M_{2}RK_{3}T\right)}}$$ (33)

$$V_{2}=\sqrt{\frac{\left(J_{2}R+M_{2}RJ_{3}T\right)\left(1-M_{1}QK_{3}T\right)+\left(K_{2}R+M_{2}RK_{3}T\right)\left(J_{1}Q+M_{1}QJ_{3}T\right)}{\left(1-M_{1}QK_{3}T\right)\left(1-M_{2}RM_{3}T\right)-\left(K_{1}Q+M_{1}QM_{3}T\right)\left(K_{2}R+M_{2}RK_{3}T\right)}}$$ (34)

$$V_{3}=\sqrt{\frac{\left(J_{3}T+J_{1}QK_{3}T\right)\left(1-K_{2}RK_{1}Q\right)+\left(M_{3}T+K_{1}QK_{3}T\right)\left(J_{2}R+K_{2}RJ_{1}Q\right)}{\left(1-M_{1}QK_{3}T\right)\left(1-K_{2}RK_{1}Q\right)-\left(M_{2}R+M_{1}RK_{2}Q\right)\left(M_{3}T+K_{1}QK_{3}T\right)}}$$ (35)

The velocity of the main channel is *V*_*m*_ = *V*_1_, the velocity of the whole floodplain is $V_{f}=\frac{V_{2}A_{2}+V_{3}A_{3}}{A_{2}+A_{3}}$, the discharge of the main channel is *Q*_*m*_ = *A*_1_ *V*_1_, that of the floodplain is *Q*_*f*_ = *A*_2_ *V*_2_ + *A*_3_ *V*_3_, and the total flow discharge is *Q* = *Q*_*m*_ + *Q*_*f*_.

When the momentum transfer between the adjoining sub-regions is ignored, namely the momentum transfer coefficient *α* = 0, the equations are the same for the single channel,

$$V_{1}=C_{1}\sqrt{R_{1}S_{0}}$$ (36)

$$V_{2}=C_{2}\sqrt{R_{2}S_{0}}$$ (37)

$$V_{3}=C_{3}\sqrt{R_{3}S_{0}}$$ (38)

## Application of the Analytical Model

The proposed model is verified by 5 runs of experimental data from Yang et al. [35] and Sun [36], in which the experiments were both under the condition of uniform flow in a compound channel with asymmetrical floodplain, and the vegetation on the floodplain were submerged and rigid cylinders. The experiment by Yang et al. [35] was carried out in a 16 m long, 0.3 m wide and 0.4 m high flume, to model the compound channel with submerged vegetation on the floodplain, in the State Key Hydraulics Laboratory of Sichuan University, China. And experiments by Sun [36] were conducted in a 12 m long and 0.306 m wide perspex tilting flume, in the hydraulics laboratory at Loughborough University, UK. Detailed experimental parameters are summarized in Table 1.

**Table 1:** Experimental conditions used to verify the proposed model.

Experiment	Run	s	B_m_ (m)	B_f_ (m)	Q (m^3^/s)	H (m)	H-h (m)	h_v_ (m)	0030	Δx (m)	Δy(m)	S_0_ (‰)
Yang et al. [35]	1	1.5	0.17	0.13	0.014	0.172	0.112	0.096	0.004	0.03	0.02	1.25
	2	1.5	0.17	0.13	0.018	0.2	0.14	0.096	0.004	0.03	0.02	1.25
	3	1.5	0.17	0.13	0.022	0.238	0.178	0.096	0.004	0.03	0.02	1.25
Sun[36]	4	1	0.55	0.365	0.104	0.27	0.12	0.11	0.06	0.12	0.24	2
	5	1	0.55	0.365	0.125	0.312	0.162	0.11	0.06	0.12	0.24	2

The proposed model is now employed to determine the velocity of each sub-region with appropriate momentum transfer coefficient for compound channel flows with submerged vegetation on the floodplain, and the results are compared with those from Eqs (36–38). Yang et al’s experiment [35] was carried out in a cement smoothing flume and Sun’s [36] in a glass flume, so the Manning coefficients are 0.011 and 0.01 respectively according to the hydraulic calculation manual [4].

As previously mentioned [40], the drag coefficient *C*_*D*_ decreases with increasing rod Reynolds number Re_*D*_ = *U*_*d*_ *D*/*υ*, and approximates to around 1.0–1.05 for Re_*D*_ up to *O* (10^3^). In this paper *C*_*D*_ was taken as 1.0.

The momentum transfer coefficients express the intense of momentum transfer between the main channel and the floodplain and the vegetation region and non-vegetation region. For non-vegetated compound channels, the study by Wang et al. [41] showed that the momentum transfer coefficient is related to the depth ratio and width ratio between the main channel and the floodplain. However, the submerged vegetation makes the turbulence stronger and obstructs the water flow, so the momentum transfer is different from that without vegetation. In this paper, *α* is valued by least square error method, as shown in Table 2. From this table, for the same series (Table 2 run 1–3 and run 4–5), the values of *α* vary in a small range, but have large differences in different series. Within one series, *α*_1_ is the biggest, while the value of *α*_2_ and *α*_3_ are not always bigger or smaller than each other. This illustrates that the momentum transfer between Regions 1 and 2 is the most intense, but those between Regions 1 and 3 and between Regions 2 and 3 may not the same under the different flow and vegetation regime.

**Table 2:** Values of momentum transfer coefficient.

Run	α_1_	α_2_	α_3_
1	0.23	0.01	0.22
2	0.28	0.01	0.27
3	0.30	0.01	0.29
4	0.07	0.05	0.01
5	0.08	0.03	0.02

By adopting the above parameters in Eqs (33–35), the velocity distribution of the compound channel with submerged rigid vegetation can be calculated. Results from the proposed equations and the errors are shown in Table 3, which shows a reasonable agreement between the measured and computed discharge of the main channel and total channel, with the biggest error of 4.05%.

**Table 3:** Calculated result and error.

Run	Measured discharge of the main channel	Measured discharge of the floodplain	Calculated discharge of the main channel	Calculated discharge of the floodplain	Error of the total discharge	Error of the main channel discharge
	Q (m^3^/s)	Q1 (m^3^/s)	Q (m^3^/s)	Q1 (m^3^/s)	(%)	(%)
1	0.0141	0.0138	0.0142	0.0137	0.64	0.58
2	0.0177	0.0163	0.0176	0.0160	0.11	1.65
3	0.0222	0.0193	0.0231	0.0196	4.01	1.78
4	0.1044	0.0996	0.1014	0.1002	2.87	0.59
5	0.1250	0.1115	0.1246	0.1136	0.32	1.85

**Fig 3:**
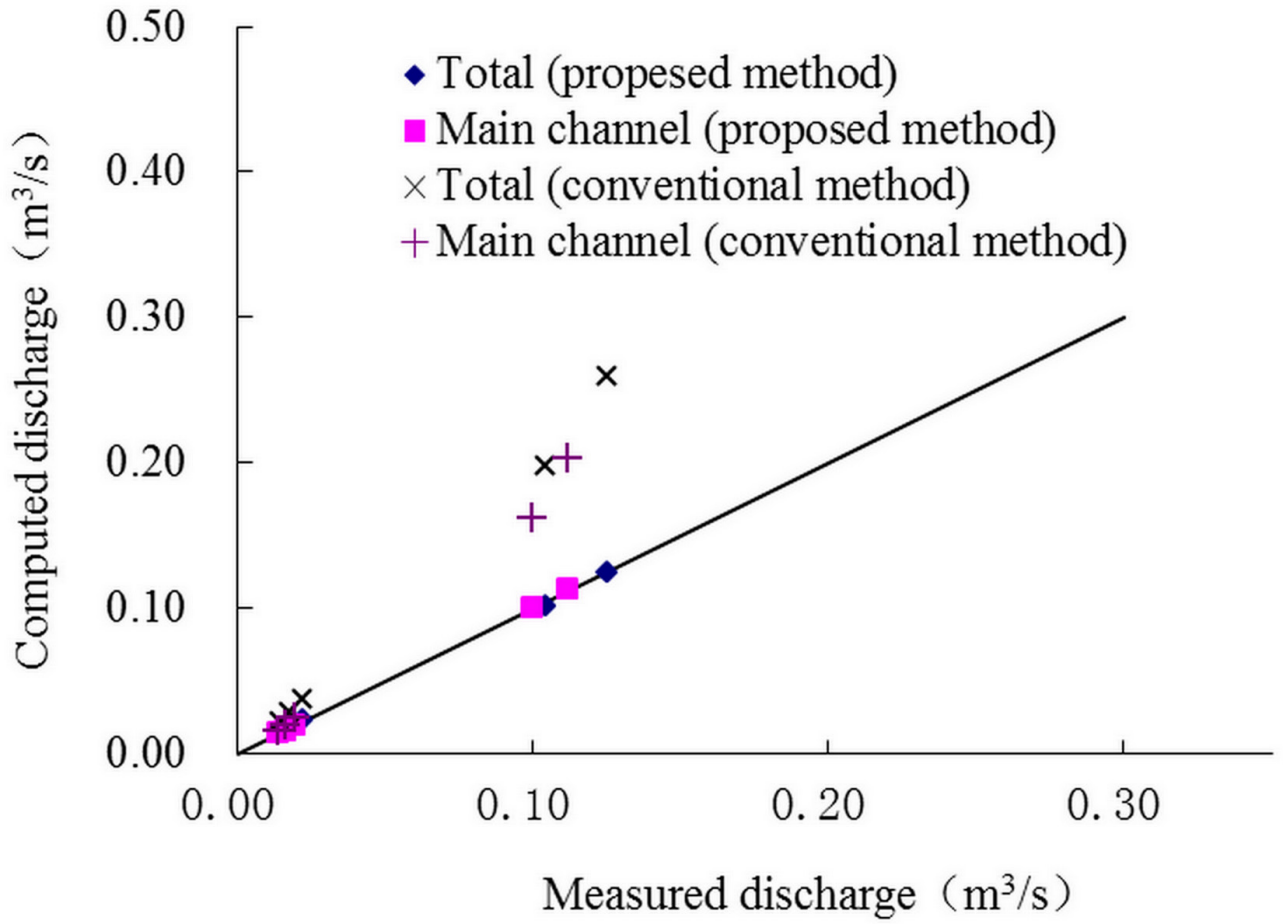
Comparison between calculation values and experimental data. Comparison between the computed and measured discharge, using the proposed and conventional methods, respectively.

**Fig 4:**
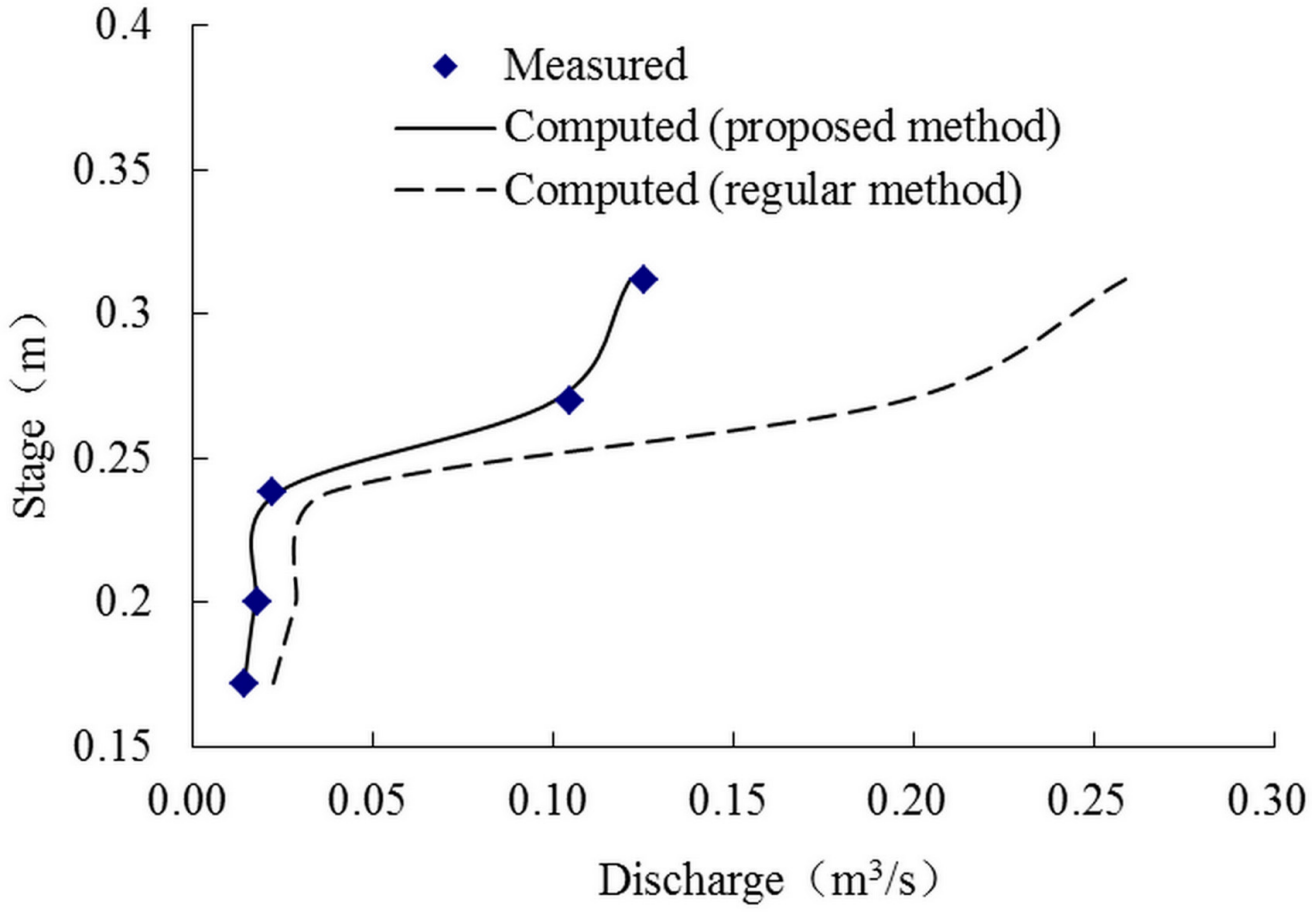
Stage-discharge curves. Comparison between the computed and measured stage-discharge relationship, using the proposed and conventional methods, respectively.

Figs 3 and 4 show the comparison between calculation values and experimental data and stage-discharge curves. It can be noted that the predictions are efficient if the momentum transfer coefficients were proper, while the predictions are much bigger than the actual values using the conventional method in which the apparent shear stress was ignored (*α* = 0), and the errors increase with the increase of water depth.

**Fig 5:**
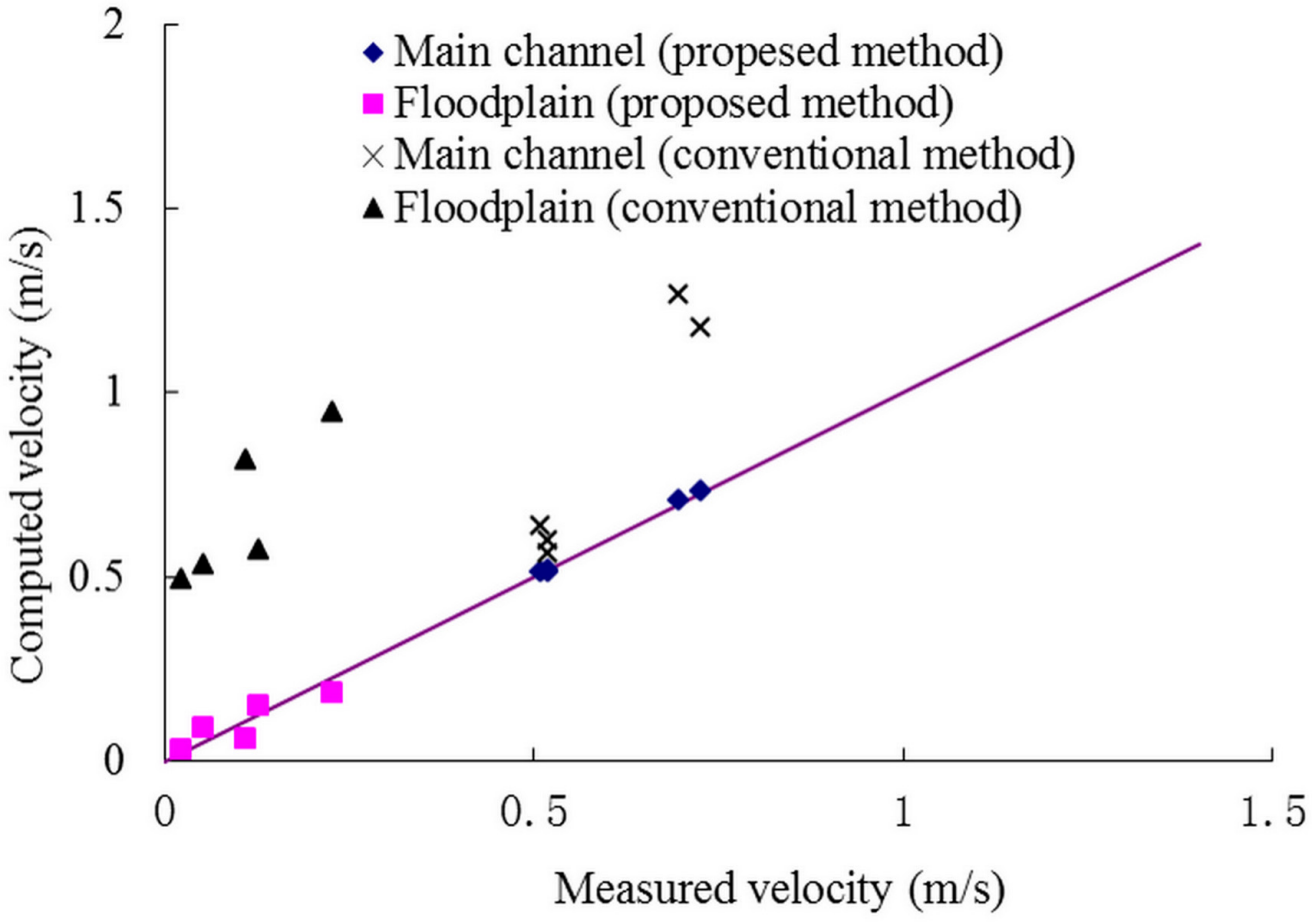
Mean velocity of the main channel and the floodplain. Comparison between the computed and measured velocities, based on the considered compound channels with vegetation emerged velocity

The mean velocity of the main channel and the floodplain is plotted in Fig 5. It can be seen that ignoring the apparent shear stress leads to great deviation from the measured data, and the error is bigger under the large discharge, especially in the floodplain region.

**Fig 6:**
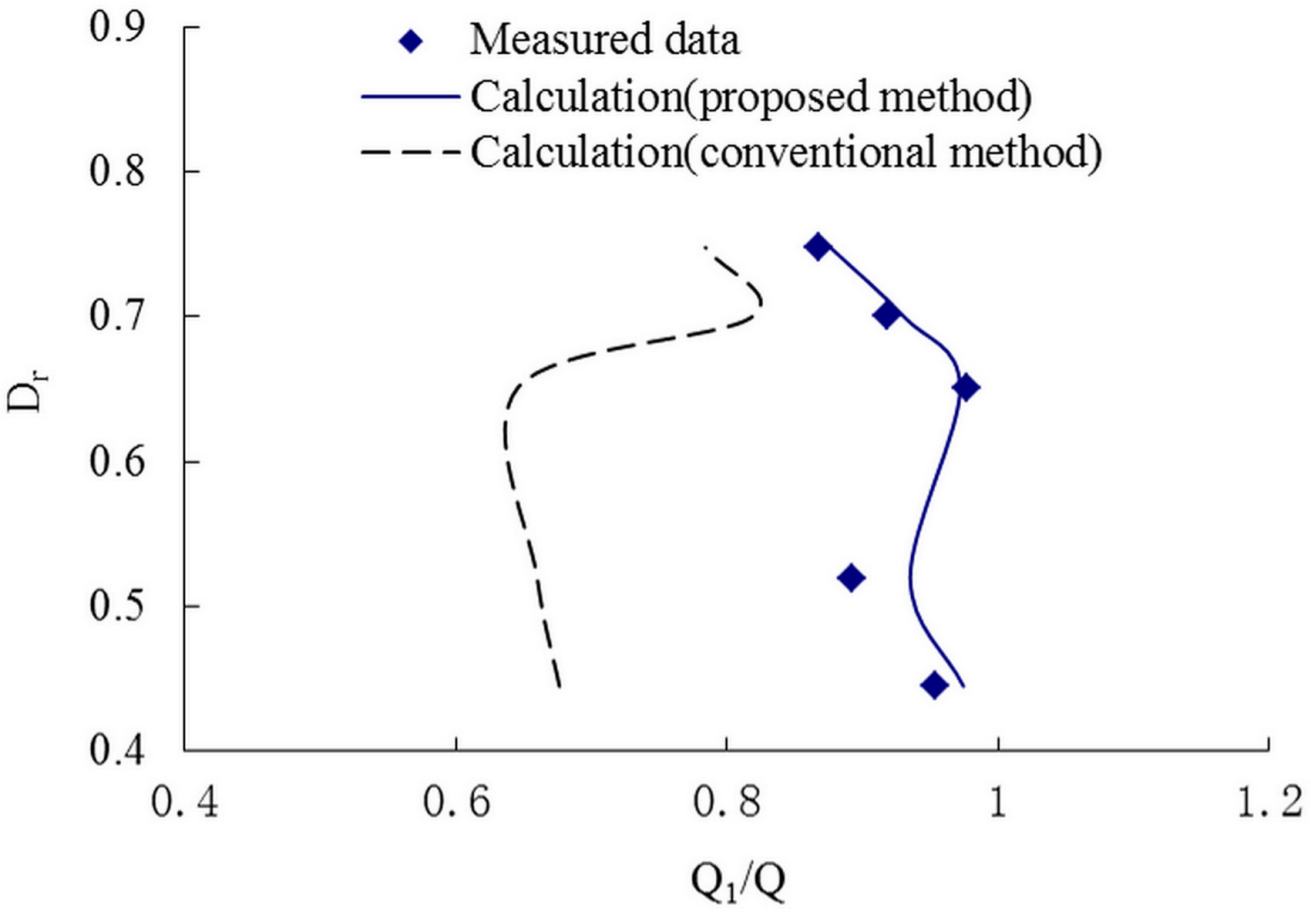
The relationship of Q1/Q and Dr. Variation of the ratio between the discharge in the main channel to that in the whole compound channel, Q1/Q, with the relative depth, Dr.

**Fig 7:**
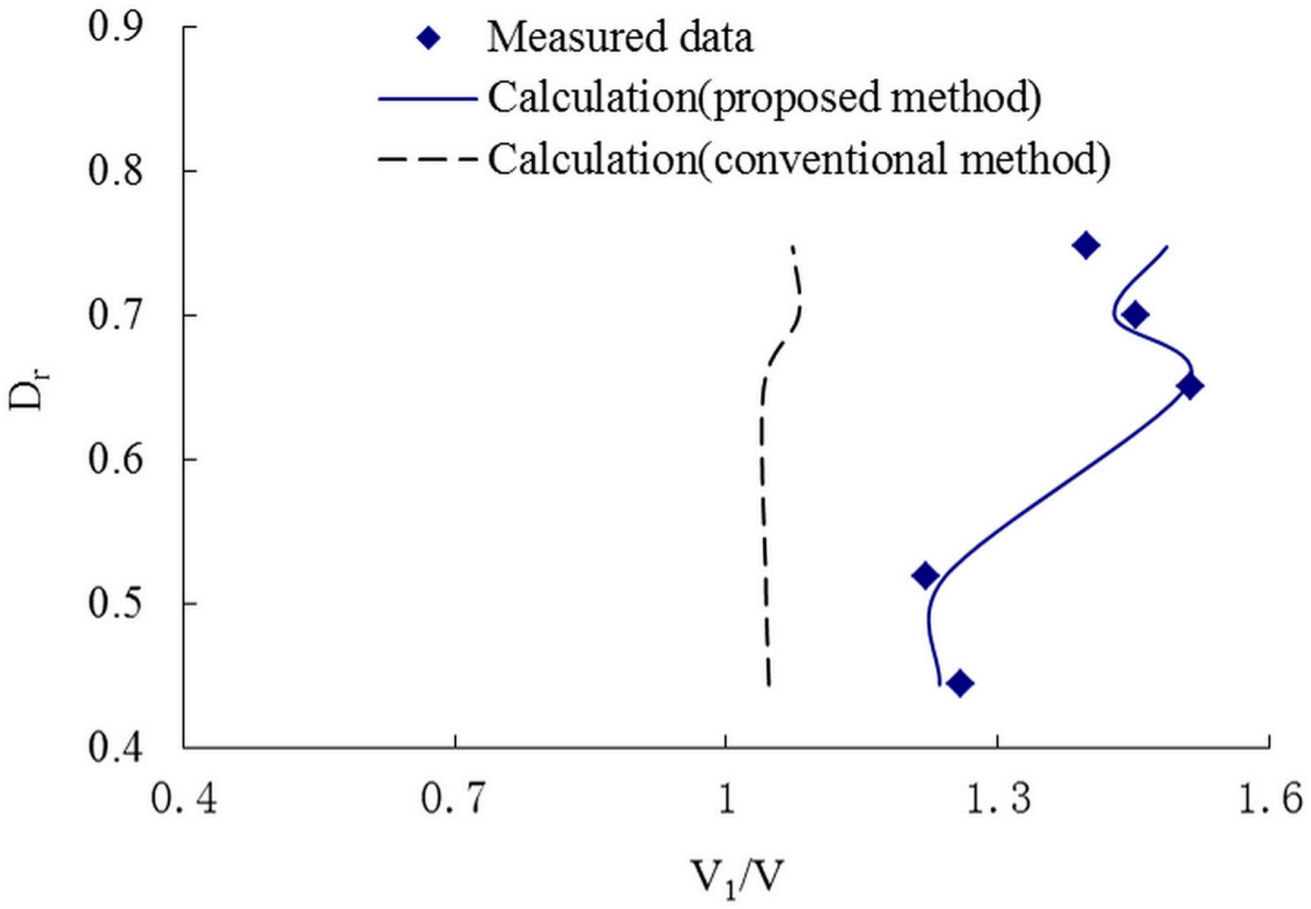
The relationship of V1/V and Dr. Variation of the ratio between the velocity in the main channel to that in the whole compound channel, V1/V, with the relative depth, Dr.

**Fig 8:**
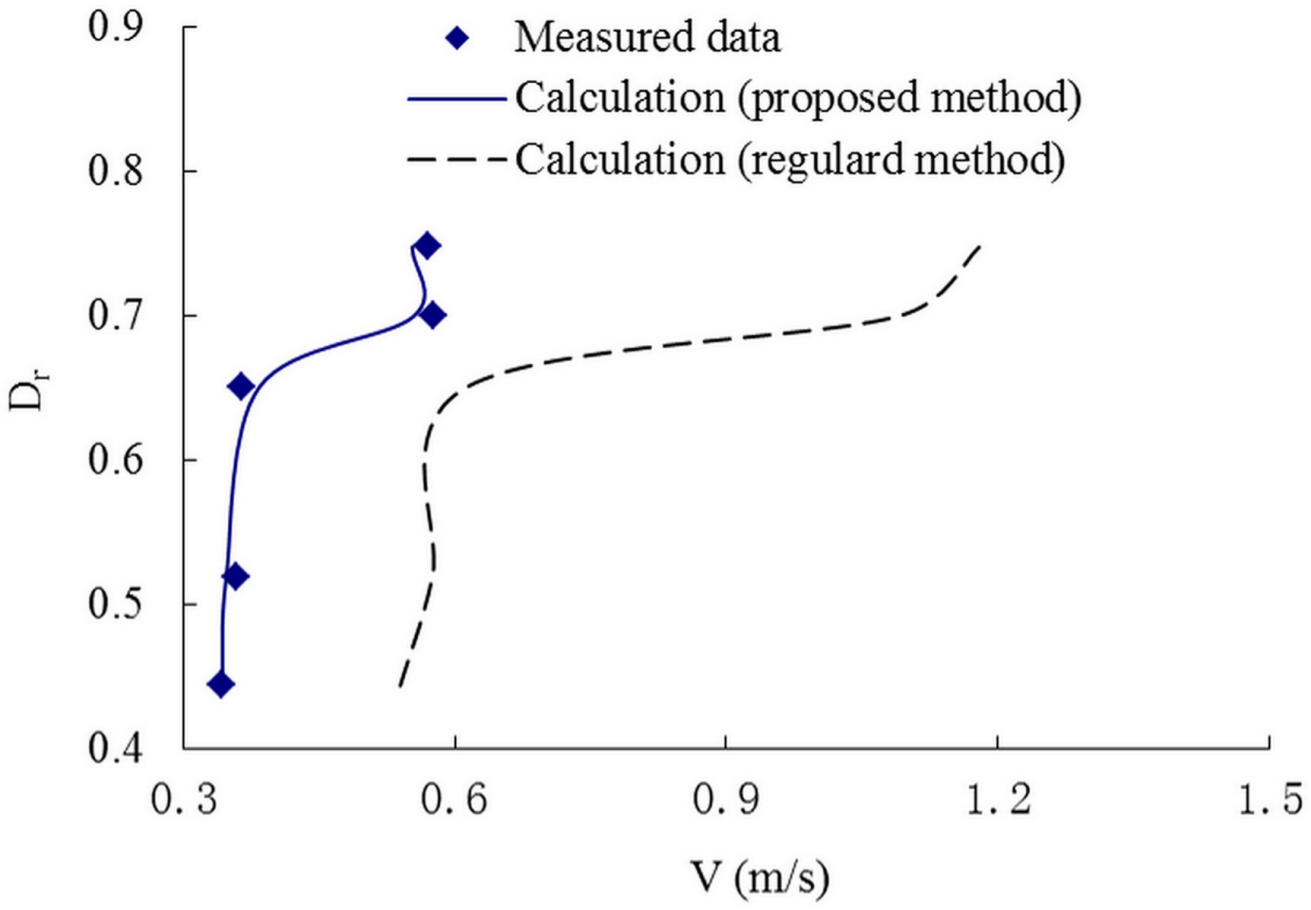
The relationship of V and Dr. Variation of the cross-sectional velocity V with the relative depth, Dr.

Figs 6–8 present the variations of the discharge ratio, Q_1_/Q and the velocity ratioV_1_/V, and the cross-sectional velocity with the relative depth, D_r_, defined as the ratio between the flow depth on the floodplain to that in the main channel. The results of discharge and velocity distribution show an excellent agreement with the measured data. But for the conventional method, even the velocity and discharge are bigger than in the proposed method. This indicates that the proposed method may improve the prediction accuracy and can be used in primary evaluation simply and conveniently.

## Comparison of Proposed Method and Other Methods and Discussion

The proposed method is in the category of momentum transfer and the apparent shear stress method (ASSM). The brief calculations of other methods are as follows,

(1) the single-channel method (SCM). The compound channel is processed as single channel. Discharge is calculated by Manning Equation,

$$Q=\frac{S_{f}^{1/2}A^{5/3}}{P^{2/3}n}$$ (39)

(2) the divided channel method (DCM). The compound channel can be divided vertically, laterally or inclined. The calculations are as Eqs (36–38).
(3) the coherence method (COHM). Ackers [13–15] proposed the 'coherence' concept to compute discharge capacity of compound channel,

$$COH=\frac{\sum_{i=1}^{i=n}\sqrt{[\sum_{i=1}^{i=n}A_{i}/\sum_{i=1}^{i=n}\left(P_{i}f_{i})\right]}}{\sum_{i=1}^{i=n}\left[A_{i}\sqrt{A_{i}/\left(P_{i}f_{i}\right)}\right]}$$ (40)

where A_i_, P_i_, f_i_ are the area, wetted perimeter and resistance coefficient for the sub-region i, n = number of floodplains + 1.

(4) comprehensive roughness method (CRM). The comprehensive roughness calculation includes Pavlovskij Method [42], Einstein-Banks Method [43], Krishnamurthy-Christensen Method [44], Lotter Method [45], and etc. If the roughness of main channel is the same as the floodplain, the computed results for the first three methods are no different as the roughness of main channel or floodplain. However as long as the hydraulic radius of each region is not equal, the computed result for Lotter Method is different from the roughness of main channel or floodplain. For this reason, the Lotter Method is chosen here. The equation is,

$$n=\frac{PR^{3/5}}{\sum_{i=1}^{i=n}\frac{P_{i}R_{i}{}^{3/5}}{n_{i}}}$$ (41)

where R_i_ is the hydraulic radius for the sub-region i.

**Fig 9:**
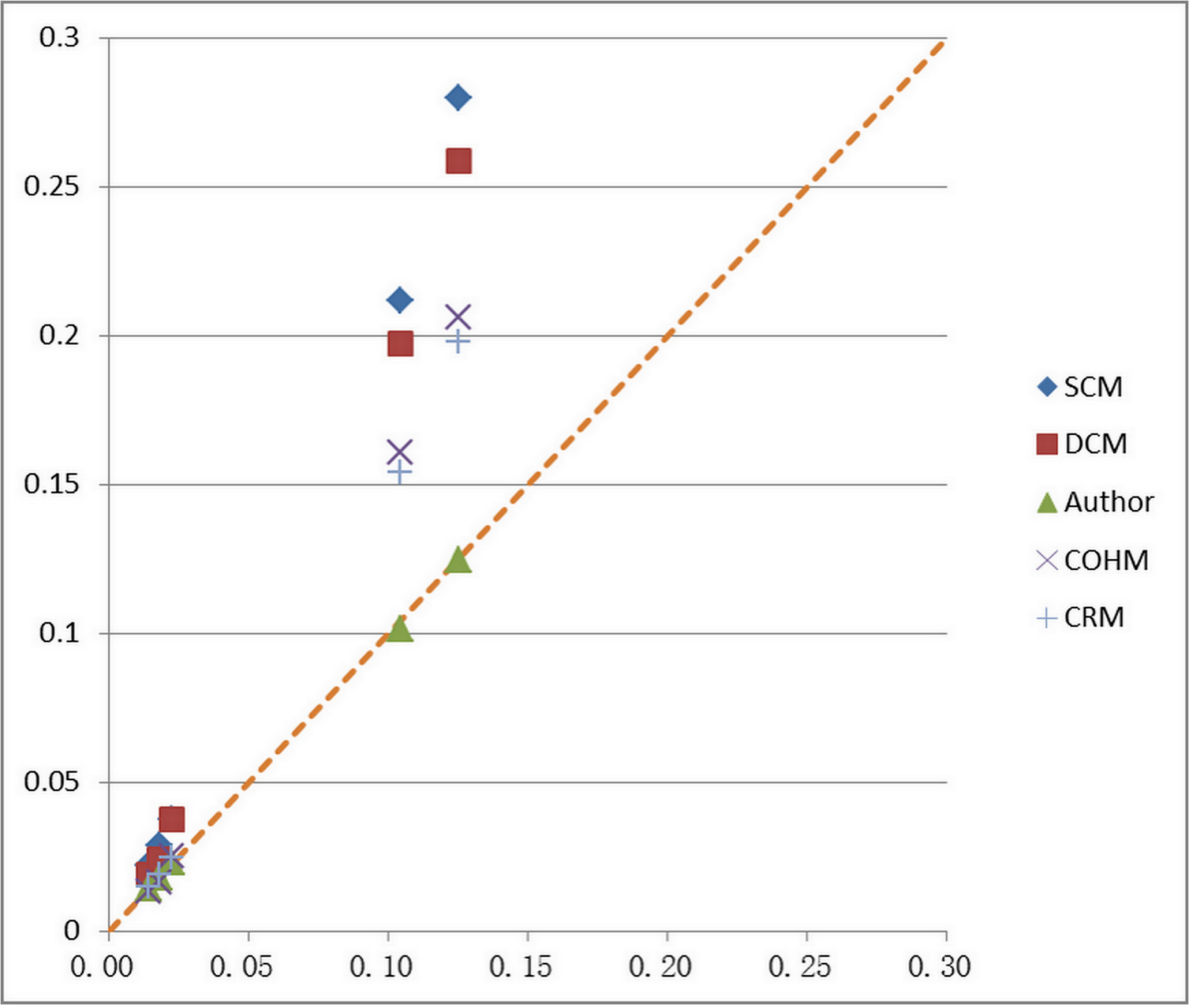
Comparison between calculative results by different methods and measured discharge: Comparison between the computed and measured discharge, using the different methods.

The results from proposed method and other methods are now compared, as shown in Fig 9 and Table 4. It turns out that the computed discharge from SCM, DCM and CRM are always higher than measured data, and the error increases with the depth. The error caused by the SCM is maximum, which can reach to 55.3%. The results from COHM are lower than measured data under shallow water depth, but higher than measured data when water depth increases. The error also increases with the depth, the maximum of which can be reach to 39.4%. The computed discharges from proposed method are not always higher or lower than measured data, and the error is not correlation with water depth. The results from the proposed method are consistent with measured discharge, which indicate the rationality of the methods.

**Table 4:** Error between discharge calculated by 6 different methods and measured discharge.

Run	H(m)	SCM(%)	DCM(%)	Author(%)	COHM(%)	CRM(%)
1	0.172	36.6	24.8	0.6	7.6	3.9
2	0.200	38.0	26.5	0.1	6.7	5.3
3	0.238	40.8	40.6	3.9	12.5	8.9
4	0.270	50.7	47.1	3.0	35.1	32.1
5	0.312	55.3	51.7	0.3	39.4	36.7

In SCM, the compound channel is regarded as single channel, which will cause remarkable error, especially under deep water depth. In CDM, when ignore the apparent shear stress, the predictions are much bigger than the actual values. The drag of submerged vegetation is not taken into account, leading to a bigger predicted value. Being applied in compound channel without vegetation, the COHM showed high accuracy [45]. The accuracy will improve if the impact of submerged vegetation on wetted perimeter and discharge cross-section was included in the calculation of ‘coherence’. The impact of vegetation and apparent shear stress are both inclusive in the proposed method, which will obtain results of high precision, with the error less than 5%. The calculation process of proposed method is simple, only momentum transfer coefficient *α* needing to be calibrated in the proposed method. Therefore the suggestion is to apply the proposed method preferentially.

## Conclusions

An analytical model is proposed for the velocity and discharge distributions in compound channels with submerged vegetation on the floodplain,based on momentum theory. By dividing into three sub-regions, i.e. the main channel region (Region 1), the floodplain region with submerged vegetation (Region 2) and floodplain region without vegetation (Region 3), the force balance relationships were established for different regions and the whole cross section. The momentum transfer coefficients were obtained by least square error method, and the velocity and discharge distribution of each region can be calculated. The comparison of the velocity and discharge distribution between the analytically predicted results and the measured data showed good agreement when the intense momentum transfer between the main channel and the floodplain and the vegetated region and non-vegetated region were taken into account via the apparent shear stress. The comparison between proposed method and other methods for predicting the conveyance capacity indicated that the results from proposed method are much more precise than others, for which the predictions are much larger than the actual values, and the errors increase with the increase of water depth. Also for its simple calculation process and parameter, we recommend a priority of proposed method in engineering calculation.
